# The Neurophysiological Impact of Experimentally-Induced Pain on Direct Muscle Spindle Afferent Response: A Scoping Review

**DOI:** 10.3389/fncel.2021.649529

**Published:** 2021-02-19

**Authors:** Carla R. Lima, Pradeep K. Sahu, Daniel F. Martins, William R. Reed

**Affiliations:** ^1^Rehabilitation Science, University of Alabama at Birmingham, Birmingham, AL, United States; ^2^Neurosciences Centre, All India Institute of Medical Sciences, New Delhi, India; ^3^Postgraduate Program in Health Sciences, Experimental Neuroscience Laboratory (LaNEx), University of Southern Santa Catarina, Palhoça, Brazil; ^4^Department of Physical Therapy, School of Health Professions, University of Alabama at Birmingham, Birmingham, AL, United States

**Keywords:** muscle spindle, pain, muscle pain, muscle, afferent, scoping review, fusimotor

## Abstract

**Background:** Musculoskeletal pain disorders are among the leading causes of years lived with disability worldwide representing a significant burden to society. Studies investigating a “nociceptive-fusimotor” relationship using experimentally-induced pain/noxious stimuli and muscle spindle afferent (MSA) response have been published over several decades. The purpose of this scoping review was to systematically identify and summarize research findings related to the impact of experimentally-induced pain or noxious stimulation on direct MSA discharge/response.

**Methods:** PubMed, Cumulative Index to Nursing and Allied Health Literature (CINAHL), Cochrane and Embase were searched from database inception to August 2020. Eligible studies were: (a) published in English; (b) clinical or pre-clinical studies; (c) original data studies; (d) included the investigation of MSA response to experimentally-induced pain or noxious stimulation; (e) included quantification of at least one direct physiological measure associated with MSA activity/response. Two-phase screening procedures were conducted by a pair of independent reviewers and data extracted from eligible studies.

**Results:** The literature search resulted in 195 articles of which 23 met inclusion criteria. Six studies (26%) were classified as clinical and 17 (74%) as pre-clinical. Two clinical studies investigated the effects of sacral dermatome pin-pricking on MSA response, while the remaining 4 studies investigated the effects of tonic muscle and/or skin pain induced by injection/infusion of hypertonic saline into the tibialis anterior muscle or subdermal tissues. In pre-clinical studies, muscle pain was induced by injection of noxious substances or the surgical removal of the meniscus at the knee joint.

**Conclusion:** Clinical studies in awake humans reported that experimentally-induced pain did not affect, or else slightly decreased MSA spontaneous discharge and/or response during weak dorsiflexor muscle contraction, thus failing to support an excitatory nociceptive-fusimotor relationship. However, a majority of pre-clinical studies indicated that ipsilateral and contralateral muscle injection of noxious substances altered MSA resting discharge and/or response to stretch predominately through static fusimotor reflex mechanisms. Methodological differences (use of anesthesia, stretch methodology, etc.) may ultimately be responsible for the discrepancies between clinical and pre-clinical findings. Additional investigative efforts are needed to reconcile these discrepancies and to clearly establish or refute the existence of nociceptive-fusimotor relationship in muscular pain.

## Introduction

Musculoskeletal pain disorders are among the leading causes of years lived with disability worldwide with low back and neck pain being listed as the most disabling musculoskeletal conditions (Brooks, 2006; Vos et al., 2017). The prevalence of musculoskeletal pain is expected to increase exponentially as individuals are living longer (Brooks, 2006; Vos et al., 2017). Considering the societal burden associated with musculoskeletal pain at the individual, community, and healthcare system levels (Brooks, 2006); there are renewed global research efforts to investigate muscle pain mechanisms particularly those pertaining to the transition from acute to chronic musculoskeletal pain.

Acute muscle pain is typically dependent on a peripheral tissue injury and/or pathology most often characterized by pain restricted to the anatomical site of injury. In the acute phase, it is generally accepted that muscle pain is generated in large part peripherally by the release of algesic and/or other chemosensitizing metabolites activating group III and IV muscle afferents (Kaufman and Rybicki, 1987; Rotto and Kaufman, 1988; Mense, 1993, 2009). However, the contribution of group III and IV afferents to the neurophysiological mechanisms responsible for the transition from acute to chronic muscle pain and increased muscle stiffness has yet to be fully elucidated.

A “nociceptive-fusimotor” or “nociceptive-proprioceptive” relationship between muscle pain and muscle spindle afferent (MSA) sensitivity has long been hypothesized to contribute to the clinical development and/or persistence of muscular pain (Korr, 1975; Johansson and Sojka, 1991). In the early 1990s, Johansson and Sojka proposed a “vicious cycle” theory where the chemosensitive activation of groups III and IV nociceptive afferents by the accumulation of excitatory metabolites in muscle, joints, and ligaments would produce reflex excitation in γ-motoneurons consequently increasing primary and/or secondary MSA responsiveness to subsequent muscle stretch while increasing homo- and heteronymous muscle stiffness. These physiological events would result in ischemia and further production of excitatory metabolites thereby creating a “vicious cycle” of muscle pain and increased MSA sensitivity, muscle stiffness and excitatory metabolite release via two positive feedback loops acting on primary and secondary MSA sensitivity resulting in motor control disturbances and/or reduced proprioceptive acuity (Johansson and Sojka, 1991). While key tenets of Johansson and Sojka's hypothesis are strongly supported by experimental evidence in multiple pre-clinical models (Jovanović et al., 1990; Johansson et al., 1993; Djupsjobacka et al., 1994, 1995a,b; Hellström et al., 2000; Hellstrom et al., 2002; Ro and Capra, 2001; Thunberg et al., 2001, 2002; Masri et al., 2005; Capra et al., 2007), this proposed nociceptive-fusimotor model for reflex driven homo- and heteronymous muscle pain and stiffness has been questioned and/or refuted by other experimental (Mense and Skeppar, 1991; Kang et al., 2001) and clinical (Birznieks et al., 2008, 2012; Fazalbhoy et al., 2013; Smith et al., 2019) studies resulting in a persistent lack of clarity on this topic. In the same year as Johansson and Sojka's presented their hypothesis, Lund et al. (1991) proposed an alternative “pain adaptation model” where physiological adaptations observed during musculoskeletal pain are related more to a protective adaptation response resulting in an inhibitory rather than a facilitatory extrafusal fiber response. Despite decades having passed from the original publication of these two muscular pain hypotheses related to the nociceptive-fusimotor relationship, controversy and discrepancies between pre-clinical and clinical research persists creating a need for re-assessment and additional avenues of investigation. This need provided the motivation for this scoping review as a means to identify and summarize past clinical and preclinical literature related to the impact of noxious stimulation and/or experimentally-induced pain on direct MSA response.

## Materials and Methods

For this scoping review, the methodological work by Arksey and O'Malley (2005) and Preferred Reporting Items for Systematic reviews and Meta-Analyses extension for scoping reviews (PRISMA-ScR) (Tricco et al., 2018) were followed. The review protocol was submitted to the “Open Science Framework” database for publication (https://osf.io/6euj8/).

### Step 1: Identifying the Research Question

The purpose of this scoping review was to identify studies and summarize findings reported in the literature related to neurophysiological effects of experimentally-induced pain and/or noxious stimulation on direct MSA activity.

### Step 2: Identifying Relevant Studies

**Figure 1:**
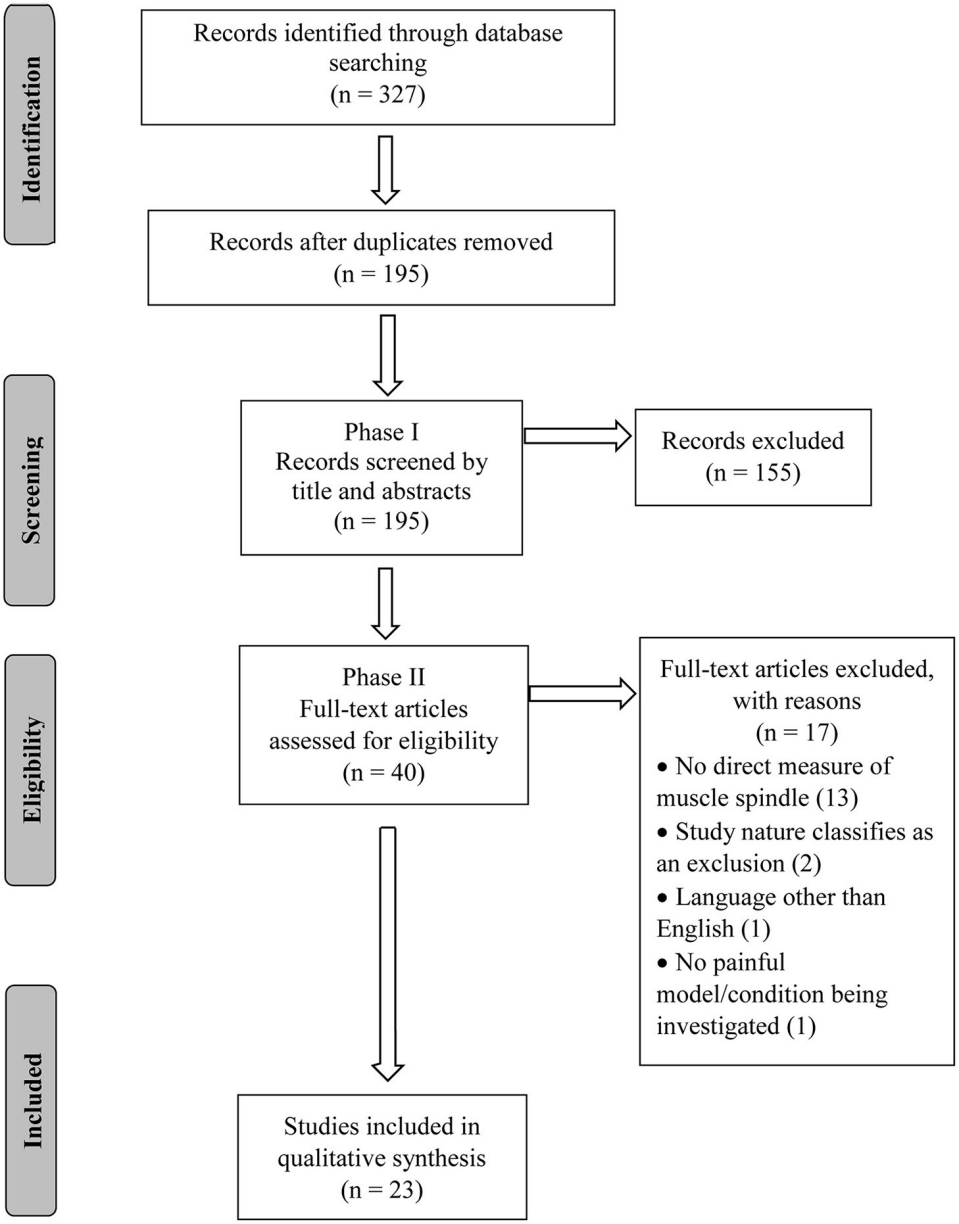
Flowchart diagram.

An appropriate search strategy was jointly developed by CRL and WRR. A combination of two search topics (pain and muscle spindle) along with their variations was used (Supplementary File A). Three electronic databases (PubMed, Cumulative Index to Nursing and Allied Health Literature—CINAHL, and Embase) were searched from their inception to August 25th, 2020. An EndNote (version X9.2, Clarivate Analytics, Boston, MA, USA) library was created, duplicates excluded and the PRISMA flow chart used to report the number of selected/excluded studies throughout the review process (Figure 1).

### Step 3: Study Selection

#### Inclusion and Exclusion Criteria

Inclusion criteria consisted of: (a) studies published in English; (b) clinical and/or pre-clinical studies; (c) original data studies; (d) included the investigation of spindle response to experimentally-induced pain or noxious stimulation; (e) included quantification of at least one direct neurophysiological measure associated with MSA discharge/response. Studies were excluded if classified as: practice guidelines, unpublished manuscripts, dissertations, reviews, expert comments, book and/or book chapters, government reports, conference proceedings, reported no direct measure of MSA discharge/response. For the purpose of this review a “direct measure” of MSA response to experimentally-induced pain or noxious stimulation was considered to be any MSA response neurophysiologically recorded directly from a peripheral nerve or dorsal nerve root. Indirect measures of muscle spindle response (such as electromyography, H-reflex studies, etc.) were excluded from this review.

#### Screening and Agreement

In the initial phase of screening, two independent reviewers (CRL, PKS) screened the titles and abstracts of the articles retrieved in the initial search. For the full-text screening, the same reviewers (CRL, PKS) independently screened the full-texts according to the pre-established inclusion and exclusion criteria. In the case of disagreement during any step of the screening process, a third reviewer (DFM) decided whether or not to include the article.

### Step 4: Data Charting

A data extraction form was developed by the research team and the following information extracted from eligible studies: author(s), year of publication, language, nature of the study (i.e., clinical/pre-clinical), target population/animal species being investigated, sample size, biological sex, purpose of the study, keywords, MSA-related response being reported, experimental pain model being investigated, and the key findings from the study. Data extraction was then performed by two authors (CRL, PKS) and verified by another author (WRR) for error minimization. Any discrepancies regarding the extracted data was resolved by a separate team member (DFM).

### Step 5: Collating, Summarizing, and Reporting the Results

Data was descriptively summarized according to the following data items:

- **Numerical and Descriptive analyses**: number of studies and trends regarding year of publication, information regarding nature of the study (clinical/pre-clinical), and biological sex.
- **Summary of findings by category:** (a) Clinical studies; (b) Pre-clinical studies.

## Results

### Numerical Analysis

**Figure 2:**
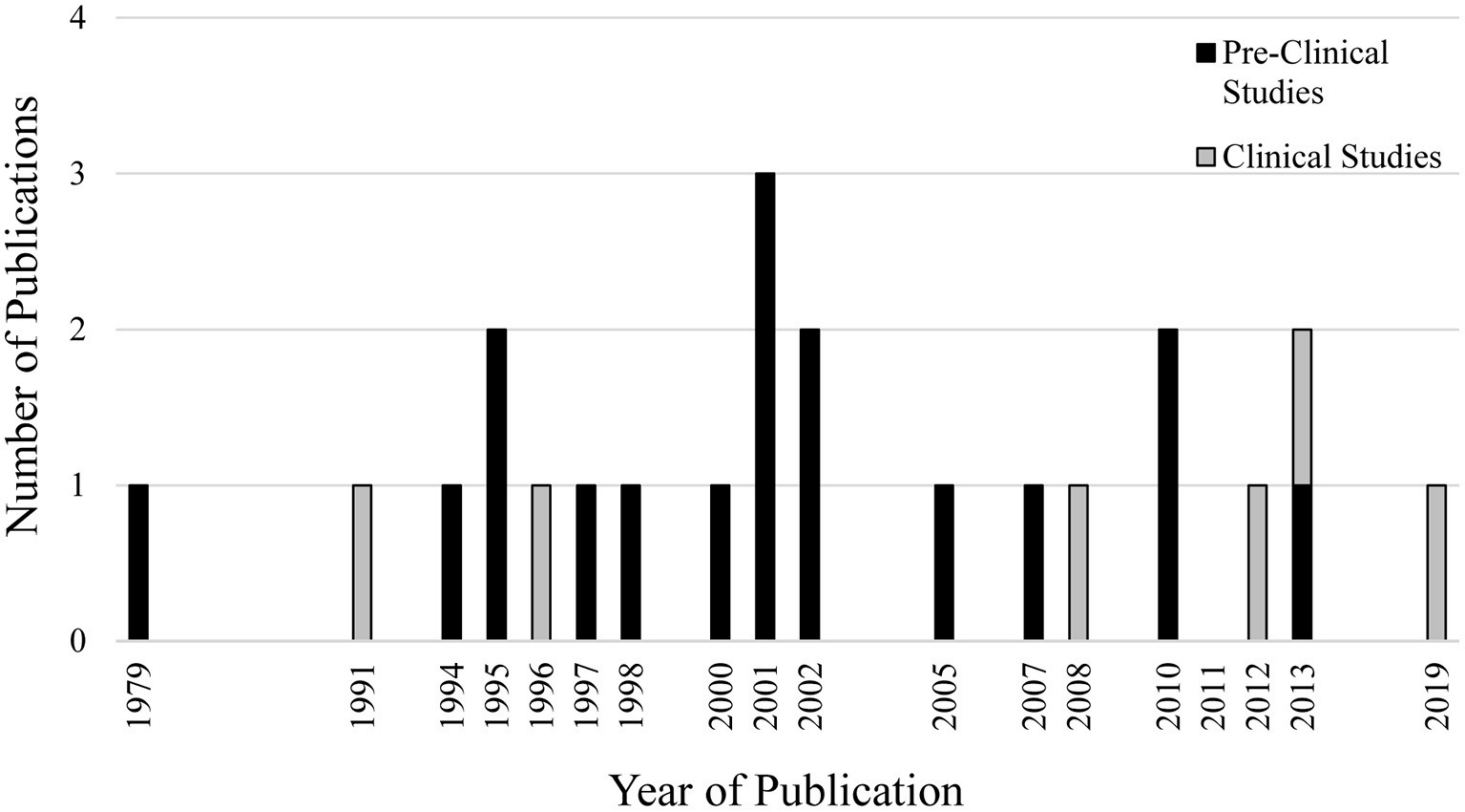
Number of publications per year related to experimentally-induced pain/noxious stimulation and muscle spindle afferent response.

The database search conducted on August 25th, 2020 resulted in 327 articles. After duplicates were removed, 195 articles had their titles and abstracts screened and 40 articles were considered relevant for full-text review and eligibility. Of these 40 articles, 17 failed to meet all eligibility requirements leaving a total of 23 articles included in this review (Figure 1). The eligible studies were published from 1979 to 2019, with 2001 being the year with the greatest number of publications (*n* = 3; Figure 2).

### Descriptive Analysis

Six studies (26%) investigated MSA response to experimentally-induced pain in humans, while 17 studies (74%) investigated MSA response to experimentally-induced pain/noxious stimulation in animal models (Figure 2). Five out of the 6 clinical studies reported the biological sex of the individuals being investigated, while only 4 out of 17 pre-clinical studies reported the biological sex of the animals.

### Clinical Studies

The 6 clinical studies investigating MSA response to experimentally-induced pain or noxious stimulation detailed information regarding the anatomical location and method of induction, the presence/absence of anesthesia, as well as the number of MSAs recorded (Supplementary File B). Two clinical studies investigated the effects of mechanical pin-prick pain to sacral structures (urinary bladder/anal sphincters) on secondary muscle spindle discharge in individuals with complete spinal cord lesions or brain death (Schalow, 1991; Schalow and Zäch, 1996). These two studies reported that noxious stimuli resulted in increases in secondary muscle spindle discharge lasting 2–3 s in duration with onset of MSA response lagging behind gamma motoneuron activity by ~ 400 ms (Schalow, 1991; Schalow and Zäch, 1996). Of note, primary MSA responses were not investigated as these spindle afferents are seldom present in the urinary bladder/anal sphincter muscles.

The remaining four clinical studies investigated the effects of experimentally-induced pain in the tibialis anterior muscle via intramuscular injection (*n* = 3) or infusion (*n* = 1) of hypertonic saline (HS; 5–7%) on MSA spontaneous discharge and/or response to weak voluntary muscle contraction (Birznieks et al., 2008, 2012; Fazalbhoy et al., 2013; Smith et al., 2019). On the whole, three of the four studies reported that intramuscular injection of HS did not significantly alter MSA resting discharge (Birznieks et al., 2012; Fazalbhoy et al., 2013; Smith et al., 2019), while one study reported a significant 6.1% mean decrease when compared to baseline (Birznieks et al., 2008). While all four HS related studies included individuals of both sexes, none of them investigated potential sex differences associated with MSA response to experimentally-induced pain. While a small number of MSAs exhibited increases in resting discharge and/or response to weak voluntary contraction (~5% maximum voluntary contraction, MVC), overall these four HS studies indicated that MSA discharge in awake humans is either not affected (no significant change) or weakly depressed following experimentally-induced muscle pain, and thereby fails to support a nociceptive-fusimotor relationship in acute muscular pain.

### Pre-clinical Studies

Seventeen studies investigated MSA response to noxious stimuli or experimentally induced pain in animal preparations under anesthesia (α-chloralose or pentobarbital). Species included: cats (11/17), rats (5/17), and monkey (1/17). Information regarding the anatomical location and method of induction, type of anesthesia, and the number of MSAs recorded can be found in Supplementary File B. Only 4/17 reported animal sex, and no pre-clinical study reported using animals of both sexes.

Seven studies investigated the effects of chemosensitization of tissues/structures within the lower extremity on MSA response (Foreman et al., 1979; Djupsjobacka et al., 1994, 1995a,b; Thunberg et al., 2002; Wu and Henry, 2010, 2013). Lower extremity structures investigated included the triceps surae (gastrocnemius, soleus), posterior biceps, semitendinosus muscles (Foreman et al., 1979; Djupsjobacka et al., 1994, 1995a,b; Thunberg et al., 2002), and knee joint (surgically induced knee osteoarthritis) (Wu and Henry, 2010, 2013). Noxious chemical injections into the lower extremity included KCl (Foreman et al., 1979; Djupsjobacka et al., 1995a), lactic acid, (Djupsjobacka et al., 1995a), arachidonic acid (Djupsjobacka et al., 1994) 5-HT (Foreman et al., 1979; Djupsjobacka et al., 1995b), bradykinin (Foreman et al., 1979; Djupsjobacka et al., 1995b), histamine (Foreman et al., 1979), 5% HS (Thunberg et al., 2002) (Supplementary File B). Thunberg et al. (2002) reported that intramuscular injection of 5% HS led to significant increases in MSA discharge (74–87%) upon sinusoidal stretching [10 successive cycles at 1 Hz, 2 mm peak-to-peak amplitude superimposed ramp-and-hold stretches [from 10 to 2 mm; maximal physiological length = 0 mm; plateau duration 15 s] of the receptor-bearing muscle and other heteronymous muscles. While the mean rate of MSA discharge increased, the average depth of modulation, respectively, decreased in a majority of MSA responses (72%) which is compatible with static fusimotor reflex action. A lesser percentage (20%) of increased MSA responses could be attributed to mixed static and dynamic fusimotor action, and the remaining 8% to inhibition of fusimotor activity (Thunberg et al., 2002). The mean duration of these effects were ~2 min and the injection of Tyrode's solution did not induce any significant changes in MSA response (Thunberg et al., 2002). For the most part, similar types and percentages of ipsilateral and contralateral lower extremity MSA responses were reported with all other noxious substance injections using the feline preparation, anesthesia (α-chloralose), and sinusoidal muscle stretching paradigm (Djupsjobacka et al., 1994, 1995a,b). Evaluating only a small number (*n* = 4) of MSAs in the monkey, Foreman et al. (1979) reported that intra-arterial injections of KCl (but not bradykinin or serotonin), significantly increased MSA discharge. These investigators also reported that despite succinylcholine's powerful excitatory action on MSAs, spinothalamic tract neurons failed to show any resultant excitatory responses (Foreman et al., 1979). Lastly, intracellular studies in the L4 dorsal root ganglion conducted by Wu and Henry (2010, 2013) reported a slowing of MSA action potential (AP) dynamics (i.e., slower AP genesis, slower AP maximum rise, and wider APs) in an experimental pain model of knee osteoarthritis.

A total of 5 pre-clinical studies looked at the effects of chemosensitization of axial (neck/trunk) tissues and/or structures on MSA response (Pedersen et al., 1997; Wenngren et al., 1998; Kang et al., 2001; Thunberg et al., 2001; Hellstrom et al., 2002). Tissues/structures investigated included the trapezius and splenius muscles (bradykinin) (Pedersen et al., 1997; Wenngren et al., 1998; Hellstrom et al., 2002), multifidus muscle (bradykinin, capsaicin) (Kang et al., 2001), and C1–C2 facet joint (bradykinin) (Thunberg et al., 2001). Four out of the 5 axial-related studies reported that noxious stimulation of axial muscle and/or joints (cervical facet, TMJ) tissue increased MSA resting discharge with a similar predominance of static fusimotor action as described in the lower extremities (Pedersen et al., 1997; Wenngren et al., 1998; Thunberg et al., 2001; Hellstrom et al., 2002). Collectively, a majority of axial findings were in agreement with findings reported in lower extremity experiments and studies were often performed by the same investigator team using the same or similar experimental set-up. However, Kang et al., using the same species (cat) and anesthesia (α-chloralose) failed to report significant changes in MSA response following bradykinin or capsaicin injection into the multifidus muscle. These investigators did not detach the ligament, but rather used ramp and hold movements of the L6 spinous process to elicit stretch of paraspinal muscles (Kang et al., 2001).

The remaining 5 pre-clinical studies investigated MSA response to chemosensitization of the masseter muscle using intramuscular injections of HS/acidic saline (Ro and Capra, 2001; Masri et al., 2005; Capra et al., 2007; Lund et al., 2010) or bradykinin (Hellstrom et al., 2002). As with a majority of the aforementioned lower extremity and axial-related MSA studies using α-chloralose anesthesia and repetitive sinusoidal stretching, noxious stimulation of the masseter muscle increased MSA discharge while decreasing the average depth of modulation suggesting a predominantly static fusimotor action (Hellstrom et al., 2002; Masri et al., 2005; Capra et al., 2007). Ro and Capra (2001) using the same species (cat) but different anesthesia (pentobarbital) reported that injection of 5% HS into the receptor-bearing muscle resulted in significant modulation (facilitation as well as reduction) in jaw opening (dynamic) and holding the jaw open (static) responses observed from 45% primary-like and 83% secondary-like MSAs. Changes in discharge activity began immediately upon HS injection (lasting 1–5 min) with pooled data indicating a subtle but significant 10–20% change of pre-injection MSA activity levels (Ro and Capra, 2001). In addition to altering MSA discharge rate, HS injection also created firing pattern irregularities (i.e., increased firing variability and altered relationships between afferent signal and muscle length) (Ro and Capra, 2001). These variability changes in MSA response offered additional support that tissue chemosensitization alters fusimotor drive to MSAs (Ro and Capra, 2001). Lastly, Lund et al. (2010) investigated whether acidic (pH = 4) saline injection into the masseter muscle led to MSA-related phenotypic and/or excitability changes that could contribute to the transition from acute to chronic muscle pain. *In vitro* recordings in the trigeminal mesencephalic nucleus (which contains the somata of masseter MSAs) demonstrated changes in several electrical properties of MSAs (spike amplitude, after hyperpolarization amplitude and duration, etc.) following acidic saline injection into the masseter muscle (Lund et al., 2010).

To summarize, pre-clinical studies reported: (a) increases in mean rate of MSA spontaneous discharge and/or during muscle stretch which was accompanied predominately by decreases depth of modulation which was thought to be related to static fusimotor action (Djupsjobacka et al., 1994, 1995a,b; Pedersen et al., 1997; Wenngren et al., 1998; Hellström et al., 2000; Hellstrom et al., 2002; Thunberg et al., 2002; Capra et al., 2007), (b) no differences between responses following noxious substance injections into ipsilateral homonymous or heteronymous muscles (Djupsjobacka et al., 1994, 1995a,b; Thunberg et al., 2002), (c) chemosensory endings in contralateral muscles were strong enough to evoke modulation of ipsilateral MSA activity (Djupsjobacka et al., 1994, 1995a,b; Pedersen et al., 1997; Wenngren et al., 1998; Capra et al., 2007), (d) typically short duration of excitatory MSA effects (1–4 min) (Djupsjobacka et al., 1994, 1995a,b; Pedersen et al., 1997; Wenngren et al., 1998; Ro and Capra, 2001; Thunberg et al., 2002; Capra et al., 2007) with the exception of lactic acid which elicited near equal excitatory and inhibitory effects lasting a mean duration of ~7 min (Djupsjobacka et al., 1995a), (e) that certain noxious substance delivery methods (i.m., i.a, i.v.) were more effective than others at eliciting MSA response (i.v. being the least effective) (Djupsjobacka et al., 1994, 1995b; Pedersen et al., 1997), (f) that deepening of anesthesia eliminated all algesic-related effects on MSA activity suggesting MSA effects were mediated through fusimotor reflexes rather than by direct chemical effects on the MSA receptors themselves (Pedersen et al., 1997; Hellström et al., 2000), and (g) an overall lack of change in muscle force, indicating that extrafusal muscle fibers remained unchanged following noxious substance injection/infusion (Djupsjobacka et al., 1995a,b; Hellstrom et al., 2002; Thunberg et al., 2002).

## Discussion

Clinical studies have long reported proprioception and motor control deficits associated with musculoskeletal pain (Revel et al., 1991; Brumagne et al., 1999; Taimela et al., 1999; Koumantakis et al., 2002) suggesting that chemosensitive group III and IV muscle afferents may directly contribute to a “nociceptive-fusimotor” relationship via their supraspinal projections and/or effects on the fusimotor-MSA system. This paradigm of a nociceptive-fusimotor relationship as it relates to muscular pain and increased stiffness has been somewhat controversial over the last three decades due to mixed pre-clinical/clinical results and thus the purpose of the present scoping review was to identify and summarize the literature regarding the direct effects of experimentally-induced pain and/or noxious stimulation on MSA response. While we acknowledge that neurophysiological insights can be gained from indirect measures of MSA response (e.g., surface electromyography, Hoffman reflex etc.), we limited this review to direct measures of MSA activity.

First, it must be acknowledged that the majority (4/6) of the clinical studies as well as a majority of pre-clinical studies (9/17) related to this search came from work predominantly conducted by two respective research teams, each publishing a progressive line of investigation using their respective experimental models. For pre-clinical studies, this entailed the use of a surgically-reduced feline preparation in which noxious substances or metabolites (HS, KCl, bradykinin, 5-HT, SCh, lactic acid, arachidonic acid, histamine, acidic saline) were injected (i.m., i.a., or i.v.) into ipsilateral and/or contralateral muscles of the lower extremity, neck and/or jaw under α-chloralose anesthesia. MSA resting discharge typically increased for brief periods (<4 min) following the noxious substance injection as this increase in MSA activity was predominately attributed to changes in static fusimotor drive. Changes in MSA response during muscle stretch were typically assessed by recording responses to 10 successive sinusoidal stretches of ipsilateral/contralateral homozygous/heterozygous muscles at a frequency of 1 Hz, 2 mm peak amplitude superimposed on the 15 s plateau of ramp-and-hold stretches (10–2 mm; maximal physiological length = 0 mm). Responses to sinusoidal stretch cycles were averaged to construct cycle histograms for which a sine function was fitted by a least mean square algorithm to provide mean rates of MSA discharge and depth of modulation [for more details see Djupsjobacka et al. (1994) and Thunberg et al. (2002)]. For clinical studies, only a single noxious substance (HS; 5–7%) was injected or infused into lower extremity homozygous/heterozygous muscles without anesthesia and in only one study was HS infusion accompanied by weak (~5% MVC) voluntary muscle contractions (Birznieks et al., 2008, 2012; Fazalbhoy et al., 2013; Smith et al., 2019). As a whole, clinical findings failed to support the majority of pre-clinical MSA findings with regard to an excitatory nociceptive-fusimotor relationship as hypothesized by Johansson and Sojka (1991). Therefore, a closer examination of experimental factors and differences that might be responsible for these discordant findings is warranted.

Several important experimental differences between the aforementioned clinical and pre-clinical studies should be noted. The most obvious is the use (or lack thereof) of anesthesia. For the most part α-chloralose was the anesthesia most utilized in pre-clinical studies (11/17), whereas clinical studies were either conducted in awake participants or failed to report the use of anesthesia. Administration of α-chloralose (25–100 mg/kg) in spinally transected and decerebrated cats has been reported to lead to slight increases in monosynaptic reflexive responses, reduction of polysynaptic reflexive responses, reduction of Renshaw cell inhibition, and inhibition (or blockage at higher doses) of the descending inhibitory action via the reticulospinal pathway (Shimamura et al., 1968; Frank and Ota, 1971). Thus, it is very possible that anesthesia acts to interfere with complex effects of descending or segmental excitatory and/or inhibitory interneuronal/fusimotor networks resulting in increased fusimotor drive to the MSA following noxious chemical injection. In two anesthetized feline preparations, subsequent L3 spinalization was performed and 7/11 MSA maintained their prior noxious substance (bradykinin, 5-HT) effects on MSA activity/response, suggesting that a considerable portion of noxious-related MSA effects are most likely segmental in nature (Djupsjobacka et al., 1995b). However, other pre-clinical evidence pointed to noxious stimulation of jaw group III and IV afferents impacting cervical muscle MSA stretch response indicating involvement of more distant inter-segmental reflexes (Hellström et al., 2000). Whether the modulation of MSA is mediated primarily through segmental, inter-segmental, or descending pathways, the potential role of anesthesia contributing to pre-clinical findings reported cannot be dismissed. In other pre-clinical studies, pentobarbital anesthesia was used (5/17), with 4/5 of these being rat studies. For the most part, pre-clinical studies using pentobarbital supported findings using α-chloralose in cats when similar experimental MSA testing procedures were performed. While pentobarbital was frequently used in cat studies to deepen the level of general anesthesia, and so as to verify the reflex-nature of noxious chemical-induced alterations; pentobarbital-resistant fusimotor activity has been reported in intercostal muscles (Sears, 1964) and suggested to be present in trunk MSA despite removal of withdrawal, pinna and corneal reflexes (Durbaba et al., 2006). Therefore, determination of the specific impact of pentobarbital, α-chloralose, and/or other anesthesia on MSA experimental response and fusimotor drive requires closer examination. Second, the 10 successive sinusoidal stretching events used in the majority of Johansson and colleagues pre-clinical work was superimposed on static stretch extending the muscle to near maximal length (Wenngren et al., 1998; Thunberg et al., 2002). This muscle stretch was strenuous enough that it prevented MSAs from falling silent during the shortening phase of the sinusoidal cycle (Wenngren et al., 1998; Thunberg et al., 2002). The near maximum degree of muscle stretch combined with the 10 successive stretches quite likely was a primary contributor to Kang et al. (2001) failure to demonstrate similar MSA changes as Johansson et al. (1993) related to muscle stretch despite the use a similar feline preparation, α-chloralose anesthesia and bradykinin/capsaicin injections into a less accessible multifidus muscle. Kang et al. (2001) indirectly stretched the multifidus muscle by loading the L6 spinous process, thereby producing a much lesser degree of total muscle stretch compared to the other feline studies. Similarly, near maximum repetitive sinusoidal stretches were not performed in any of the clinical studies. Only one clinical study involved voluntary changes in muscle length following HS infusion, and these voluntary ankle dorsiflexor contractions were only at weak forces (~5% MVC) suggesting minimal muscle stretch at best (Smith et al., 2019). In addition to the aforementioned points, it should be noted that frequently the depth of modulation reported was small in nature (Ro and Capra, 2001), and that different muscles with distinct physiological functions (i.e., neck, back, and/or jaw muscles) may respond differently to noxious substance delivery and/or to repetitive stretch than hindlimb muscles. For example, clear anatomical and organizational MSA differences are well-documented between neck and lower extremity MSAs (Richmond and Abrahams, 1979; Richmond and Bakker, 1982), and significant differences in MSA response were noted to occur between splenius and trapezius muscles following bradykinin injections in pre-clinical preparations (Pedersen et al., 1997; Wenngren et al., 1998). While, HS preferentially excites small diameter muscle fibers with minimal effects on MSA themselves (Paintal, 1960) (suggesting a potential impact of HS on fusimotor drive) (Ro and Capra, 2001; Thunberg et al., 2002), HS failed to significantly increase MSA spontaneous discharge or MSA response during isometric ankle dorsiflexion in humans (Birznieks et al., 2008; Fazalbhoy et al., 2013; Smith et al., 2019). However, HS did result in greater MSA discharge irregularities/variability in both pre-clinical (Ro and Capra, 2001) and clinical (Smith et al., 2019) studies.

In a majority of anesthetized pre-clinical studies, immediate increases (>70%) in MSA discharge in response to noxious chemical injection (HS, KCl, bradykinin, 5-HT, lactic acid, arachidonic acid, histamine, acidic saline) were found to have mean durations of excitatory effects typically lasting <4 min and usually peaking within 1–2 min after injection. This result from the acute delivery of noxious chemicals stands in contrast to clinical studies using slow infusion delivery of HS lasting 45–60 min in awake humans (Fazalbhoy et al., 2013; Smith et al., 2019). Infusion rates of HS were adjusted to create a state of perceived pain of 5–6 out of 10, and yet no excitatory effects on MSA discharge were demonstrated to occur either in relaxed muscle (Fazalbhoy et al., 2013) or during weak voluntary contractions (Smith et al., 2019). Adequate exposure of MSAs in the tibialis anterior muscle to HS should not have been a concern due to the prolonged period of infusion and the level of perceived pain experienced. Much of the increase in MSA discharge in anesthetized pre-clinical studies was explained by reflex action of muscle nociceptors on static fusimotor neurons. Of note, significant resting fusimotor outflow primarily in static γ-motorneurons has been reported in decerebrate cats (Matthews and Stein, 1969), while being absent or negligible in relaxed human muscles (Burke, 1981; Nordh et al., 1983). However, in awake HS human studies involving voluntary muscle contractions (which involves a descending excitatory drive co-activating fusimotor activity), no increase in MSA discharge was noted (Smith et al., 2019). While the human studies had admittedly smaller sample sizes, other possible explanations for these discrepancies likely involve the consequences of anesthesia either decreasing descending drive to spinal circuits or uncovering nociceptor-fusimotor spinal reflexes normally suppressed in the awake state. The lesser degree of muscle stretch performed in human studies, and/or the possibility of direct or indirect activation of MSA receptor endings by different noxious chemicals acting on spindle annulospiral endings which contain synaptic-like vesicles expressing the glutamate transporter VGLUT1 (indicating that they can release glutamate) (Wu et al., 2004; Bewick et al., 2005; Lund et al., 2010) also may have contributed to the discrepancies. It has been reported that many small-caliber nerve fibers in close proximity to muscle spindle annulospiral endings are immunoreactive for calcitonin gene-related peptide (CGRP) and P2X3 (and to a lesser extent Substance P, and TRPV1) (Lund et al., 2010). It is unclear the role these nociceptive markers played in the pre-clinical MSA response. Other noted differences that could contribute to the discrepancy between pre-clinical and clinical study findings, include the fact that human MSA mean background firing rates are much lower (~10 Hz) compared to animals (9–40 Hz) (Burke et al., 1979; Gregory et al., 1991; Proske et al., 1991; Macefield, 2013; Macefield and Knellwolf, 2018), as well as animals demonstrating greater independent fusimotor control than humans (Vallbo, 1971; Loeb, 1984; Prochazka et al., 1985; Macefield and Knellwolf, 2018). To clarify the role of these potential contributors to pre-clinical and clinical discordant findings, additional investigation will be required.

### Limitations

Significant experimental limitations are associated with both clinical and preclinical studies. These limitations include: (1) the physical invasiveness required to directly record MSA response often limited the number of afferents recorded in a majority of clinical studies, (2) the inability to determine the specific role anesthesia plays on descending and/or segmental excitatory and/or inhibitory circuitry related to fusimotor drive (it might be of value to repeat human tonic muscle pain MSA studies while participants are under anesthesia as a means to better address the role and importance of anesthesia), (3) the inability to detach ligaments from their insertion to allow near maximal stretch of the muscle in human studies, and (4) differences in delivery methodology (i.m., i.a., i.v; injection, infusion) and the inherent chemical properties, physiological interactions, and/or concentrations of noxious substances used (HS, KCl, NaCl, bradykinin, etc.) used to experimentally induce pain or provide noxious stimulation.

Limitations specifically associated with this scoping review include: (1) limiting publications in English only thus, potentially reducing the number of studies being retrieved from the literature search; (2) excluding articles looking at indirect measures of muscle spindle responsiveness (e.g., surface electromyography, Hoffman reflex, etc.) which narrowed the focus and reduced the total number of studies retrieved from the literature search; and (3) although this review primarily focused on the investigation of physiological outcomes, the analysis of pain severity and/or behavioral outcomes could potentially expand on the appraisal of different experimental preparations.

### Future Directions

To address some of these experimental questions and/or research limitations, additional pre-clinical and/or clinical research using different types of anesthesia which act through different physiological mechanisms, as well as the use of additional noxious substances that might produce more sustained experimental pain or noxious effects are needed. For example, pain-related substances like nerve growth factor (NGF) which has been temporally associated with the mechanical hyperalgesia and identified as a major contributor to delayed-onset muscle soreness may prove beneficial. Injection of neurotrophins and/or other metabolites naturally associated with sustained muscle pain or injury could bring further clarity to a potential nociceptor-fusimotor relationship. Another identified gap in this literature review was the lack of comparisons between biological sexes. Most of the studies reviewed either failed to report the biological sex, investigated only one sex, and/or failed to have adequate sample sizes to make biological sex comparisons. Sex-linked muscle spindle numerical variation has been identified in abdominal muscles of some animals (Bortolami and Martini, 1970). The mean number of intrafusal fibers per spindle pole as well as the percentage of multiple bag fibers were also found to be higher in males compared to females in cat superficial lumbrical muscles (Decorte et al., 1990). Differences in spindle composition between animals of different sex raise interesting questions with regard to muscle pain and possible functional consequences, in much the same way as anatomical and organizational differences do between spindles in neck and lower extremity musculature (Richmond and Abrahams, 1979; Richmond and Bakker, 1982). Considering the biological, mechanistic and subjectively reported sex differences in musculoskeletal pain reported in the literature (Mogil and Bailey, 2010; Sorge et al., 2015; Sorge and Totsch, 2017), we suggest that future clinical and pre-clinical studies, investigate possible sex-related differences in MSA response to experimentally-induced pain. In addition, greater investigation of more chronic musculoskeletal pain models like experiments conducted by Wu and Henry (Wu and Henry, 2010, 2013) would be tremendously beneficial in clarifying the effects of experimentally-induced pain on MSA activity.

## Conclusions

Results from the clinical studies in this review suggest that MSA response in humans is unlikely affected by acute or prolonged experimentally-induced pain, at least as it related to use of 5–7% hypertonic saline. Pre-clinical studies using different models and anesthesia suggest the existence of a nociceptor-fusimotor relationship, however the impact of anesthesia, fusimotor control, small-caliber fibers near the annulospiral endings immunoreactive for nociceptive markers, and repetitive sinusoidal stretch protocols need to be clarified in experimental preparations. Lastly, none of the studies reviewed here investigate possible biological sex differences in MSA response to experimentally-induced muscular pain and this area specifically needs to be addressed in future experiments.

## Data Availability Statement

The original contributions generated for the study are included in the article/Supplementary Material, further inquiries can be directed to the corresponding author/s.

## Author Contributions

CL and WR contributed to conception and study design, data extraction, data analyses, manuscript writing, and revision. PS contributed to data extraction, data analyses, and manuscript revision. DM contributed to conception, manuscript writing, and revision. All authors read and approved the submitted version.

## Conflict of Interest

The authors declare that the research was conducted in the absence of any commercial or financial relationships that could be construed as a potential conflict of interest.

## References

[B1] Arksey H. O'Malley L. (2005). Scoping studies: towards a methodological framework. Intl. J. Soc. Res. Methodol. 8, 19–32. 10.1080/1364557032000119616

[B2] Bewick G. S. Reid B. Richardson C. Banks R. W. (2005). Autogenic modulation of mechanoreceptor excitability by glutamate release from synaptic-like vesicles: evidence from the rat muscle spindle primary sensory ending. J. Physiol. 562, 381–394. 10.1113/jphysiol.2004.074799 15528245 PMC1665510

[B3] Birznieks I. Boonstra T. W. Macefield V. G. (2012). Modulation of human muscle spindle discharge by arterial pulsations–functional effects and consequences. PLoS ONE 7:e35091. 10.1371/journal.pone.0035091 22529975 PMC3328488

[B4] Birznieks I. Burton A. R. Macefield V. G. (2008). The effects of experimental muscle and skin pain on the static stretch sensitivity of human muscle spindles in relaxed leg muscles. J. Physiol. 586, 2713–2723. 10.1113/jphysiol.2008.151746 18403422 PMC2536575

[B5] Bortolami R. Martini E. (1970). Sex-linked muscle spindle numerical variations in the abdominal muscles of some mammals. Sperimentale 120, 115–129. 4260591

[B6] Brooks P. M. (2006). The burden of musculoskeletal disease–a global perspective. Clin. Rheumatol. 25, 778–781. 10.1007/s10067-006-0240-3 16609823

[B7] Brumagne S. Lysens R. Spaepen A. (1999). Lumbosacral repositioning accuracy in standing posture: a combined electrogoniometric and videographic evaluation. Clin. Biomechan. 14, 361–363. 10.1016/S0268-0033(98)00086-2 10521615

[B8] Burke D. (1981). The activity of human muscle spindle endings in normal motor behavior. Intl. Rev. Physiol. 25, 91–126. 6451598

[B9] Burke D. Skuse N. F. Stuart D. G. (1979). The regularity of muscle spindle discharge in man. J. Physiol. 291, 277–290. 10.1113/jphysiol.1979.sp012812 158085 PMC1280900

[B10] Capra N. F. Hisley C. K. Masri R. M. (2007). The influence of pain on masseter spindle afferent discharge. Arch. Oral. Biol. 52, 387–390. 10.1016/j.archoralbio.2006.10.011 17126284 PMC1868482

[B11] Decorte L. Emonet-Dénand F. Harker D. W. Laporte Y. (1990). Individual differences in multiple-bag spindles of cat superficial lumbrical muscles. J. Anatomy 169, 1–12. 2143502 PMC1256952

[B12] Djupsjobacka M. Johansson H. Bergenheim M. (1994). Influences on the gamma-muscle-spindle system from muscle afferents stimulated by increased intramuscular concentrations of arachidonic acid. Brain Res. 663, 293–302. 10.1016/0006-8993(94)91276-9 7874514

[B13] Djupsjobacka M. Johansson H. Bergenheim M. Sjolander P. (1995a). Influences on the gamma-muscle-spindle system from contralateral muscle afferents stimulated by KCl and lactic acid. Neurosci. Res. 21, 301–309. 10.1016/0168-0102(94)00864-C 7777220

[B14] Djupsjobacka M. Johansson H. Bergenheim M. Wenngren B. I. (1995b). Influences on the gamma-muscle spindle system from muscle afferents stimulated by increased intramuscular concentrations of bradykinin and 5-HT. Neurosci. Res. 22, 325–333. 7478296 10.1016/0168-0102(95)00906-a

[B15] Durbaba R. Taylor A. Ellaway P. H. Rawlinson S. (2006). Classification of longissimus lumborum muscle spindle afferents in the anaesthetized cat. J. Physiol. 571, 489–498. 10.1113/jphysiol.2005.102731 16410280 PMC1796785

[B16] Fazalbhoy A. Macefield V. G. Birznieks I. (2013). Tonic muscle pain does not increase fusimotor drive to human leg muscles: implications for chronic muscle pain. Exp. Physiol. 98, 1125–1132. 10.1113/expphysiol.2012.071670 23417691

[B17] Foreman R. D. Schmidt R. F. Willis W. D. (1979). Effects of mechanical and chemical stimulation of fine muscle afferents upon primate spinothalamic tract cells. J. Physiol. 286, 215–231. 10.1113/jphysiol.1979.sp012615 108391 PMC1281567

[B18] Frank G. B. Ota M. (1971). Blockade of the reticulospinal inhibitory pathway by anaesthetic agents. Br. J Pharmacol. 42, 328–342. 10.1111/j.1476-5381.1971.tb07117.x 4934743 PMC1665657

[B19] Gregory J. E. Morgan D. L. Proske U. (1991). Two kinds of resting discharge in cat muscle spindles. J. Neurophysiol. 66, 602–612. 10.1152/jn.1991.66.2.602 1774588

[B20] Hellstrom F. Thunberg J. Bergenheim M. Sjolander P. Djupsjobacka M. Johansson H. (2002). Increased intra-articular concentration of bradykinin in the temporomandibular joint changes the sensitivity of muscle spindles in dorsal neck muscles in the cat. Neurosci. Res. 42, 91–99. 10.1016/S0168-0102(01)00307-8 11849728

[B21] Hellström F. Thunberg J. Bergenheim M. Sjölander P. Pedersen J. Johansson H. (2000). Elevated intramuscular concentration of bradykinin in jaw muscle increases the fusimotor drive to neck muscles in the cat. J. Dent. Res. 79, 1815–1822. 10.1177/00220345000790101401 11078000

[B22] Johansson H. Djupsjöbacka M. Sjölander P. (1993). Influences on the gamma-muscle spindle system from muscle afferents stimulated by KCl and lactic acid. Neurosci. Res. 16, 49–57. 10.1016/0168-0102(93)90008-E 8387164

[B23] Johansson H. Sojka P. (1991). Pathophysiological mechanisms involved in genesis and spread of muscular tension in occupational muscle pain and in chronic musculoskeletal pain syndromes: a hypothesis. Med. Hypotheses 35, 196–203. 10.1016/0306-9877(91)90233-O 1943863

[B24] Jovanović K. Anastasijević R. Vuco J. (1990). Reflex effects on gamma fusimotor neurones of chemically induced discharges in small-diameter muscle afferents in decerebrate cats. Brain Res. 521, 89–94. 10.1016/0006-8993(90)91528-O 2207680

[B25] Kang Y. M. Wheeler J. D. Pickar J. G. (2001). Stimulation of chemosensitive afferents from multifidus muscle does not sensitize multifidus muscle spindles to vertebral loads in the lumbar spine of the cat. Spine 26, 1528–1536. 10.1097/00007632-200107150-00005 11462081

[B26] Kaufman M. P. Rybicki K. J. (1987). Discharge properties of group III and IV muscle afferents: their responses to mechanical and metabolic stimuli. Circ. Res. 61, I60–65. 3652404

[B27] Korr I. M. (1975). Proprioceptors and somatic dysfunction. J. Am. Osteopath. Assoc. 74, 638–650. 124754

[B28] Koumantakis G. A. Winstanley J. Oldham J. A. (2002). Thoracolumbar proprioception in individuals with and without low back pain: intratester reliability, clinical applicability, and validity. J. Ortho. Sports Phys. Ther. 32, 327–335. 10.2519/jospt.2002.32.7.327 12113467

[B29] Loeb G. E. (1984). The control and responses of mammalian muscle spindles during normally executed motor tasks. Exerc. Sport Sci. Rev. 12, 157–204. 10.1249/00003677-198401000-00008 6234174

[B30] Lund J. P. Donga R. Widmer C. G. Stohler C. S. (1991). The pain-adaptation model: a discussion of the relationship between chronic musculoskeletal pain and motor activity. Can. J. Physiol. Pharmacol. 69, 683–694. 10.1139/y91-102 1863921

[B31] Lund J. P. Sadeghi S. Athanassiadis T. Caram Salas N. Auclair F. Thivierge B.. (2010). Assessment of the potential role of muscle spindle mechanoreceptor afferents in chronic muscle pain in the rat masseter muscle. PLoS ONE 5:e11131. 10.1371/journal.pone.0011131 20559566 PMC2886111

[B32] Macefield V. G. (2013). Discharge rates and discharge variability of muscle spindle afferents in human chronic spinal cord injury. Clin. Neurophysiol. 124, 114–119. 10.1016/j.clinph.2012.05.015 22727338

[B33] Macefield V. G. Knellwolf T. P. (2018). Functional properties of human muscle spindles. J. Neurophysiol. 120, 452–467. 10.1152/jn.00071.2018 29668385

[B34] Masri R. Ro J. Y. Capra N. (2005). The effect of experimental muscle pain on the amplitude and velocity sensitivity of jaw closing muscle spindle afferents. Brain Res. 1050, 138–147. 10.1016/j.brainres.2005.05.039 15982645

[B35] Matthews P. B. Stein R. B. (1969). The regularity of primary and secondary muscle spindle afferent discharges. J. Physiol. 202, 59–82. 10.1113/jphysiol.1969.sp008795 4238988 PMC1351465

[B36] Mense S. (1993). Nociception from skeletal muscle in relation to clinical muscle pain. Pain 54, 241–289. 10.1016/0304-3959(93)90027-M 8233542

[B37] Mense S. (2009). Algesic agents exciting muscle nociceptors. Exp. Brain Res. 196, 89–100. 10.1007/s00221-008-1674-4 19139871

[B38] Mense S. Skeppar P. (1991). Discharge behaviour of feline gamma-motoneurones following induction of an artificial myositis. Pain 46, 201–210. 10.1016/0304-3959(91)90077-B 1749644

[B39] Mogil J. S. Bailey A. L. (2010). Sex and gender differences in pain and analgesia. Prog. Brain Res. 186, 141–157. 10.1016/B978-0-444-53630-3.00009-9 21094890

[B40] Nordh E. Hulliger M. Vallbo A. B. (1983). The variability of inter-spike intervals of human spindle afferents in relaxed muscles. Brain Res. 271, 89–99. 10.1016/0006-8993(83)91367-7 6883123

[B41] Paintal A. S. (1960). Functional analysis of group III afferent fibres of mammalian muscles. J. Physiol. 152, 250–270. 10.1113/jphysiol.1960.sp006486 14429833 PMC1363314

[B42] Pedersen J. Sjölander P. Wenngren B. I. Johansson H. (1997). Increased intramuscular concentration of bradykinin increases the static fusimotor drive to muscle spindles in neck muscles of the cat. Pain 70, 83–91. 10.1016/S0304-3959(96)03305-2 9106812

[B43] Prochazka A. Hulliger M. Zangger P. Appenteng K. (1985). 'Fusimotor set': new evidence for alpha-independent control of gamma-motoneurones during movement in the awake cat. Brain Res. 339, 136–140. 10.1016/0006-8993(85)90632-8 3161585

[B44] Proske U. Gregory J. E. Morgan D. L. (1991). Where in the muscle spindle is the resting discharge generated? Exp. Physiol. 76, 777–785. 10.1113/expphysiol.1991.sp003543 1835843

[B45] Revel M. Andre-Deshays C. Minguet M. (1991). Cervicocephalic kinesthetic sensibility in patients with cervical pain. Arch. Phys. Med. Rehabil. 72, 288–291. 2009044

[B46] Richmond F. J. Abrahams V. C. (1979). Physiological properties of muscle spindles in dorsal neck muscles of the cat. J. Neurophysiol. 42, 604–617. 10.1152/jn.1979.42.2.604 154558

[B47] Richmond F. J. Bakker D. A. (1982). Anatomical organization and sensory receptor content of soft tissues surrounding upper cervical vertebrae in the cat. J. Neurophysiol. 48, 49–61. 10.1152/jn.1982.48.1.49 6214617

[B48] Ro J. Y. Capra N. F. (2001). Modulation of jaw muscle spindle afferent activity following intramuscular injections with hypertonic saline. Pain 92, 117–127. 10.1016/S0304-3959(00)00477-2 11323133

[B49] Rotto D. M. Kaufman M. P. (1988). Effect of metabolic products of muscular contraction on discharge of group III and IV afferents. J. Appl. Physiol. 64, 2306–2313. 10.1152/jappl.1988.64.6.2306 3136123

[B50] Schalow G. (1991). Coactivity of secondary spindle afferents and alpha 2, alpha 3, gamma 1 and gamma 2-motoneurons innervating anal and urinary bladder sphincters in humans. Electromyogr. Clin. Neurophysiol. 31, 223–241. 1831752

[B51] Schalow G. Zäch G. A. (1996). Reflex stimulation of continuously oscillatory firing alpha and gamma-motoneurons in patients with spinal cord lesion. Gen. Physiol. Biophys. 15(Suppl. 1), 75–93. 8934198

[B52] Sears T. A. (1964). Efferent discharges in alpha and fusimotor fibres of intercostal nerves of the cat. J. Physiol. 174, 295–315. 10.1113/jphysiol.1964.sp007488 14244124 PMC1368954

[B53] Shimamura M. Yamauchi T. Aoki M. (1968). Effects of chloralose anesthesia on spinal reflexes. Jpn. J. Physiol. 18, 788–797. 10.2170/jjphysiol.18.788 5305292

[B54] Smith L. J. Macefield V. G. Birznieks I. Burton A. R. (2019). Effects of tonic muscle pain on fusimotor control of human muscle spindles during isometric ankle dorsiflexion. J. Neurophysiol. 121, 1143–1149. 10.1152/jn.00862.2018 30699044

[B55] Sorge R. E. Mapplebeck J. C. Rosen S. Beggs S. Taves S. Alexander J. K.. (2015). Different immune cells mediate mechanical pain hypersensitivity in male and female mice. Nat. Neurosci. 18, 1081–1083. 10.1038/nn.4053 26120961 PMC4772157

[B56] Sorge R. E. Totsch S. K. (2017). Sex differences in pain. J. Neurosci. Res. 95, 1271–1281. 10.1002/jnr.23841 27452349

[B57] Taimela S. Kankaanpää M. Luoto S. (1999). The effect of lumbar fatigue on the ability to sense a change in lumbar position. A controlled study. Spine 24, 1322–1327. 10.1097/00007632-199907010-00009 10404574

[B58] Thunberg J. Hellstrom F. Sjolander P. Bergenheim M. Wenngren B. Johansson H. (2001). Influences on the fusimotor-muscle spindle system from chemosensitive nerve endings in cervical facet joints in the cat: possible implications for whiplash induced disorders. Pain 91, 15–22. 10.1016/S0304-3959(00)00415-2 11240074

[B59] Thunberg J. Ljubisavljevic M. Djupsjöbacka M. Johansson H. (2002). Effects on the fusimotor-muscle spindle system induced by intramuscular injections of hypertonic saline. Exp. Brain Res. 142, 319–326. 10.1007/s00221-001-0941-4 11819039

[B60] Tricco A. C. Lillie E. Zarin W. O'Brien K. K. Colquhoun H. Levac D.. (2018). PRISMA Extension for Scoping Reviews (PRISMA-ScR): checklist and explanation. Ann. Internal Med. 169, 467–473. 10.7326/M18-0850 30178033

[B61] Vallbo A. B. (1971). Muscle spindle response at the onset of isometric voluntary contractions in man. Time difference between fusimotor and skeletomotor effects. J. Physiol. 218, 405–431. 10.1113/jphysiol.1971.sp009625 4256547 PMC1331803

[B62] Vos T. Abajobir A. A. Abate K. H. Abbafati C. Abbas K. M. Abd-Allah F.. (2017). Global, regional, and national incidence, prevalence, and years lived with disability for 328 diseases and injuries for 195 countries, 1990-2016: a systematic analysis for the global burden of disease study 2016. Lancet 390, 1211–1259. 10.1016/S0140-6736(17)32154-2 28919117 PMC5605509

[B63] Wenngren B. I. Pedersen J. Sjölander P. Bergenheim M. Johansson H. (1998). Bradykinin and muscle stretch alter contralateral cat neck muscle spindle output. Neurosci. Res. 32, 119–129. 10.1016/S0168-0102(98)00074-1 9858019

[B64] Wu Q. Henry J. L. (2010). Changes in Abeta non-nociceptive primary sensory neurons in a rat model of osteoarthritis pain. Mol. Pain 6, 37. 10.1186/1744-8069-6-37 20594346 PMC2908067

[B65] Wu Q. Henry J. L. (2013). Peripheral drive in Aα/β-fiber neurons is altered in a rat model of osteoarthritis: changes in following frequency and recovery from inactivation. J. Pain Res. 6, 207–221. 10.2147/JPR.S40445 23671396 PMC3650889

[B66] Wu S. X. Koshimizu Y. Feng Y. P. Okamoto K. Fujiyama F. Hioki H.. (2004). Vesicular glutamate transporter immunoreactivity in the central and peripheral endings of muscle-spindle afferents. Brain Res. 1011, 247–251. 10.1016/j.brainres.2004.03.047 15157812

