# Supplementary material for: The Neurophysiological Impact of Experimentally-Induced Pain on Direct Muscle Spindle Afferent Response: A Scoping Review
Source: Front Cell Neurosci. 2021 Feb 19;15:649529. doi: 10.3389/fncel.2021.649529 (PMC7933477; doi:10.3389/fncel.2021.649529)
Supplement: Supplementary file 1 [file Table_1.DOCX]

**Supplementary File A. Search Terms**

(pain OR "muscle pain" OR "musculoskeletal pain" OR "muscle nocicept*" OR "muscle hyperalgesia" OR "muscle hypersensitivity" OR "muscle sensitivity" OR "muscular nocicept*" OR "muscular hyperalgesia" OR "muscular hypersensitivity" OR "muscular sensitivity") AND ("muscle spindle" OR "muscle spindles" OR "Bag1 fiber" OR "Bag2 fiber" OR "nuclear chain fiber" OR "nuclear bag fiber" OR "gamma motoneurons" OR "gamma-motoneurons" OR "γ-motoneurons" OR "gamma motor system" OR "gamma-motor system" OR "spindle afferent" OR "type Ia afferent" OR "type II afferent")
