# Supplementary material for: The Neurophysiological Impact of Experimentally-Induced Pain on Direct Muscle Spindle Afferent Response: A Scoping Review
Source: Front Cell Neurosci. 2021 Feb 19;15:649529. doi: 10.3389/fncel.2021.649529 (PMC7933477; doi:10.3389/fncel.2021.649529)
Supplement: Supplementary file 2 [file Table_2.docx]

| **Clinical Studies** | | | | | | |
| --- | --- | --- | --- | --- | --- | --- |
| **Author (year)** | **Population** | **Sample Size (Sex)** | **Location of pain stimulus or injection (substance, amount)** | **Anesthesia (Y/N). If yes, substance (amount)** | **Spindles afferents (N)** | **Key Findings** |
| Schalow, 1991 | Humans  (brain death) | N=9 (sex nr*) | Mechanical - pin-prick of sacral dermatomes (urinary bladder/anal sphincters & adjacent tissues) | nr* | nr* | Pin-prick pain transiently ↑ secondary MSA discharge ~400ms after an ↑ in α-motoneuron activity with 90% of delay originating in muscle spindles |
| Schalow et al., 1996 | Humans (complete spinal cord lesion) | N=9 (M+F quantity nr*) | Mechanical - pin-prick of sacral dermatomes (urinary bladder/anal sphincters & adjacent tissues) | nr* | nr* | Pin-prick pain led to a longer and stronger reduction of secondary MSA response |
| Birznieks et al., 2008 | Healthy  Humans | N=15 (11M/4F) | Intramuscular (I.M.) - tibialis anterior or sub-dermally (5% hypertonic saline - HS, 0.5 ml injection) | N | 13/19  analyzed for muscle & skin pain | HS-induced muscle pain ↓ MSA spontaneous discharge by 6.1%  (no change [n=6], decrease [n=7]) |
| Birznieks et al., 2012 | Healthy  Humans | N=40 (24M/16F) | I.M. - tibialis anterior or sub-dermally (5% HS, 0.5 ml injection) | N | 12/75 analyzed for muscle pain  13/75 skin pain | HS-induced muscle or skin pain but did not significantly change spontaneous MSA discharge (muscle pain caused small decrease [9.9 to 9.4 imp/s] in resting discharge |
| Fazalbhoy et al., 2013 | Healthy  Humans | N=15 (10M/5F) | I.M. - tibialis anterior infusion (7% HS, 250 μl/min for 10-50min) | N | 15 | HS-induced pain but did not significantly change spindle afferent discharge |
| Smith et al., 2019 | Healthy  Humans | N=13 (9M/4F) | I.M. - tibialis anterior infusion (5% HS, nr*) | N | 10  (n=6 contractions tested in absence/ presence of pain) | HS-induced pain did not significantly change spindle afferent discharge during weak voluntary contractions (~5MVC) |

Note: nr*= not reported; M= males; F= females; Y/N= yes or no; ms= milliseconds; I.M.= intramuscular; ↑= increase; ↓= decrease; MSA= muscle spindle afferent; HS= hypertonic saline; imp/s= impulses per second; ml= milliliters; μl/min= microliters per minute; min= minute; MVC= maximum voluntary contraction.

| **Pre-Clinical Studies** | | | | | | |
| --- | --- | --- | --- | --- | --- | --- |
| **Author (year)** | **Species** | **Sample Size**  **(Sex)** | **Location of injection (substance, amount)** | **Anesthesia (Y/N). If yes, substance (amount).** | **Spindles afferents (N)** | **Key Findings** |
| Foreman et al., 1979 | Monkey | N=20  (sex nr*) | Ipsilateral- sural artery (BK 23 μg/ 5-HT 135μg/ KCl 3.8mg/ histamine 180μg/SCh 164μg); 0.1-0.3ml  I.M.- Triceps Surae/Achilles Tendon (6% NaCl, 0.1-0.3ml). | Y, α-chloralose (80 mg/kg) followed by an infusion of sodium pentobarbital (2 mg/kg per h). | 4 | Varied response depending on fiber type/substance:  BK, 5-HT, and Histamine - weak excitation in MSA discharge;  KCl and SCh - strong excitation of muscle spindle afferent discharge |
| Djupsjobacka  et al.,1994 | Cat | N=5  (sex nr*) | Ipsilateral- sural artery;  contralateral -brachial vein  1mg/ml, 0.3-1.0ml, rate of 1.0ml/min | Y, α-chloralose (60 mg/kg) | 36 | Ipsi- & Contralateral Arachidonic acid ↑ MSA firing rate predominately via static or mixed (static & dynamic) fusimotor drive |
| Djupsjobacka  et al.,1995a | Cat | N=7  (sex nr*) | Contralateral - Sural & Femoral arteries  Contralateral - brachial vein (KCl 200-400 mM; Latic Acid 20-50 mM; 1.0ml at rate of 1.0 ml/min | Y, α-chloralose (70 mg/kg) | 42 | Contralateral KCl (excitatory MSA response) & lactic acid (inhibitory MSA response) via static (lactic acid), dynamic (KCl), and/or mixed fusimotor drive |
| Djupsjobacka  et al.,1995b | Cat | N=6  (sex nr*) | Ipsilateral -sural artery  Contralateral - brachial vein (BK 9–100 mg/ml; 5-HT 25–150 mg/ml); 0.5-1.0 ml at rate of 1.0 ml/min | Y, α-chloralose (60 mg/kg) | 47 | Ipsi- & contralateral BK increased MSA discharge via static, dynamic, and mixed fusimotor drive, while 5-HT injections increased MSA responses via static fusimotor drive  Responses persisted after cord transection thus segmentally governed |
| Pedersen et al., 1997 | Cat | N=5  (sex nr*) | Contralateral I.M. - Splenius & Trapezius (BK 0.5 ml at 6–86 μg/ml) | Y, α-chloralose (60 mg/kg) | 29 | BK ↑ stretch response of contralateral MSA via static fusimotor drive |
| Wenngren et al., 1998 | Cat | N=5  (sex nr*) | Contralateral I.M.- Splenius & Trapezius (BK 6-86 μg/ml, 0.5 ml total volume) | Y, α-chloralose (60 mg/kg). | 20 | Contralateral BK ↑ MSA response via ↑ static fusimotor drive |
| Hellstrom et al., 2000 | Cat | N=7  (sex nr*) | Ipsilateral I.M.- Masseter (BK 0.5 ml, 12.5-50 μg/ml) | Y, α-chloralose (60 mg/kg). | 23 | BK ↑ MSA response via static (and to lessor degree mixed) via fusimotor drive |
| Kang et al., 2001 | Cat | N=25  (sex nr*) | Subfascial or I.M. - Multifidus 0.5 ml (BK 5–100 mg, capsaicin 100 μg–100 mg) | Y, α-chloralose (35 mg/kg) or midcollicular decerebration (n=7) | 25 | Neither BK or Capsaicin significantly affected MSA discharge in α-chloralose or decerebrate preparations |
| Ro et al., 2001 | Cat | N=9  (sex nr*) | I.M.- Masseter (5% HS, 250 μl) | Y, pentobarbital sodium (38 mg/kg) | 35 | HS facilitated and reduced mean firing rates of MSA during jaw movements |
| Thunberg et al., 2001 | Cat | N=7  (sex nr*) | Intra-articular - C1/C2 Facet Joint (BK 12-50μg/ml) | Y, α-chloralose (60 mg/kg). | 25 | BK ↑ MSA activity via static (and to lesser degree mixed) fusimotor drive |
| Hellstrom et al., 2002 | Cat | N=8  (sex nr*) | Ipsilateral Intra-articular– TMJ joint (BK 12.5–50 μg/ml, 0.1ml) | Y, α-chloralose (60 mg/kg). | 26 | BK ↑ MSA firing rate (10 static; 2 mixed) and ↓ MSA firing rate (3 mixed) fusimotor drive intersegmental reflexes |
| Thunberg et al., 2002 | Cat | N=6  (sex nr*) | I.M.- Gastrocnemius & Posterior Biceps (5% HS, 0.4–0.6 ml) | Y, α-chloralose (60 mg/kg). | 42 | HS ↑ MSA discharge rate (predominantly static & to a lesser degree mixed) fusimotor drive |
| Masri et al., 2005 | Rat | N=nr*  (sex *nr) | Ipsi-, contralateral, & distant I.M. – Masseter & hindlimb (5% HS, 100 μl) | Y, pentobarbital sodium (45 mg/kg) | 45 | HS facilitated & reduced MSA discharge (distant injection had no effect) via predominate static fusimotor drive |
| Capra et al., 2007 | Rat | N=nr*  (M) | I.M.- Masseter (5% HS, 100 μl) | Y, pentobarbital sodium (45 mg/kg) | 43 | HS ↑ MSA discharge via static fusimotor drive with little effect on dynamic sensitivity |
| Lund et al., 2010 | Rat | Total N=214 (behavior, cfos, immunofluorescence  & electrophysiology)  n=72 (muscle spindle-related *in vitro* recordings)  (M) | I.M.- Masseter (acidic saline [0.9% NaCl, 20 μl, pH 4.0]) | *In vitro*  recordings | 308 | Acidic saline: ↑ MSA excitability (initiation of ectopic action potentials) and MSA peripheral terminals contain VGLUT1, CGRP, SubP, P2X3 receptors |
| Wu et al., 2010 | Rat | Total  N= 81  (n=35 muscle spindle-related  recordings)  (F) | Mechanical - Knee OA - Surgical removal medial meniscus and partial cut of anterior cruciate ligament | Y, pacuronium (1 mg/kg) and supplemental pentobarbital (20 mg/kg) | 47 | Chronic OA produced action potential changes including slower genesis, slower rise time/rise rate, wider action potential duration at base in intracellular DRG MSA *in vivo* |
| Wu et al., 2013 | Rat | N=nr*  (F) | Mechanical - Knee OA - Surgical removal medial meniscus and partial cut of anterior cruciate ligament | Y, pacuronium (1 mg/kg) and supplemental pentobarbital (20 mg/kg) | 91 | Chronic OA produced shorter refractory intervals in paired pulse stimulation and greater maximum following frequency in train stimulation among spindle afferents *in vivo* |

Note: nr*= not reported; M= males; F= females; Y/N= yes or no; MSA= muscle spindle afferent; BK= bradykinin; 5-HT= 5-hydroxytryptamine; KCl= potassium chloride; SCh= succinylcholine; I.M.= intramuscular; HS= hypertonic saline; C1/C2= cervical 1-2 joint; TMJ= temporomandibular joint; NaCL= Sodium chloride; pH= potential of hydrogen; VGLUT1= vesicular glutamate transporter 1; CGRP= calcitonin gene related peptide; SubP= substance P; P2X3= P2X purinoceptor 3; OA= osteoarthritis; DRG= dorsal root ganglion; ↑= increase; μg= micrograms; ml= milliliters; mg= micrograms; kg= kilograms; mM= millimolar; ml= milliliters; μl= microliters; min= minute;
